# The clinical and the laboratory autoimmunologist: Where do we stand?

**DOI:** 10.1186/s13317-020-00133-1

**Published:** 2020-07-07

**Authors:** Renato Tozzoli, Nicola Bizzaro

**Affiliations:** 1 Laboratorio di Patologia Clinica, Ospedale San Antonio, Tolmezzo, Italy; 2 Azienda Sanitaria Universitaria Integrata di Udine, Udine, Italy; 3 Dipartimento di Medicina di Laboratorio, Presidio Ospedaliero S. Maria degli Angeli, Pordenone, Italy

**Keywords:** Autoimmunologist, Autoimmunology, Test interpretation, Consultation

As highlighted in many medical reports from industrialized countries, autoimmune diseases (AIDs) are increasing in number (almost 100 distinct AIDs have been classified to date) and in epidemiology (5–10% of individuals worldwide are affected by an AID) [1, 2]. In the United States, AIDs are collectively estimated to affect from 5 to 8% of the population or 15 to 20 million individuals [3] and are cumulatively the third cause of illness and mortality in humans [2, 4]. Consequently, many efforts are produced by health systems for prevention, early diagnosis and treatment of autoimmune diseases.

This ‘autoimmune pandemic’ has led to the creation of a new medical discipline, autoimmunology [5, 6], which is now considered a new subcategory either of clinical immunology or of internal medicine [7], or even a distinct branch [8], due to the increased awareness of its complexity and the consequent need of highly specialized competence. That autoimmunology is an emerging discipline is also documented in medical literature by the growth of dedicated journals and by a progressively increasing annual publication rate characterized by defined bibliometric and scientometric properties [8].

The progressive evolution of knowledge on the role of autoantibodies as indicators of the pathogenesis, diagnosis and prognosis of autoimmune diseases [9] has led to an increase in antibody test requests to clinical laboratories. At the same time, the technological progress and the activity of the biomedical industry in the sector has produced new analytical methods and new automated instrumental platforms allowing a faster and more precise and accurate execution of autoantibody tests, configuring a profound revolution in autoimmune diagnostics. Therefore, the organizational structure of clinical laboratories has progressively changed to respond more and more efficiently to clinical needs through the consolidation of autoantibody tests in larger laboratories with higher operational capacity and the transition of production lines from academic laboratories to general laboratories [10].

In view of these changes, starting around ten years ago, we and others highlighted the need for the creation and recognition of a new specialist, the autoimmunologist [5, 6, 11], including two types of specialists in this subdiscipline: the clinical autoimmunologist and the laboratory autoimmunologist [12].

## The clinical autoimmunologist: a promise still partially missed

In 2009, Vasconcelos [13], in response to the (for him pleonastic) question: ‘Do we need autoimmune disease units in hospitals?’, was very emphatically in favour of the creation of a transversal competency to which the different physicians working in the area (i.e. rheumatologists, nephrologists, gastroenterologists, endocrinologists, etc.) could consult, in order to share expertise and harmonize clinical practice in autoimmunology. At the time, this type of professional figure and the presence of specialized units for clinical management and research of AIDs in big hospitals were extremely rare in the world, with the notable exception of the Zabludowicz Center for Autoimmune Disease at the Sheba Medical Center in Tel Hashomer, Israel (first director: Yehuda Shoenfeld); the Department of Autoimmunology at the Statens Serum Institut of Copenhagen, Denmark (first director: Allan Wiik); and the Autoimmune Disease Research Center at the Institute for Basic Biomedical Sciences, John Hopkins University, Baltimore (first director: Noel Rose). Despite the efforts of these pioneers, the vision of autoimmune diseases as one, albeit articulated, unique entity is still limited, probably as a consequence of a diffuse separated clinical practice in hospitals, with a low level of collaboration between the different types of clinical specialists.

However, some significant initiatives have been implemented over time. Some years ago, the ceaseless activity of Yehuda Shoenfeld and Allan Wiik led to the creation of the European Autoimmunity Standardization Initiative (EASI) [14, 15] and more recently of the Centers of Excellence in Autoimmunity (ACE) in order to provide cutting-edge research, care, and teaching, within the wider scope of autoimmune diseases [5]. To our knowledge, centers dedicated to autoimmune diseases with multidisciplinary teams are present mainly in the United States and include the ACE departments at the Feinstein Institute for Medical Research, Manhasset (New York); at the Massachusetts General Hospital, Boston; at the Oklahoma Medical Research Foundation, Oklahoma; at the University of Michigan, Ann Arbor; at the University of Colorado, Denver; at the University of California, San Francisco; and at the University of Pennsylvania, Philadelphia. In South America, the Center for Autoimmune Disease Research (CREA) is active at the Mederi Hospital in Bogota, Colombia. In Europe some centers are present in Spain (Autoimmune Disease Units at the San Cecilio Hospital in Granada and at the CIMA Hospital in Barcelona) and in Italy (the Immuno Center at the Humanitas Clinical Institute in Milan and the Unit of Autoimmune Diseases at Federico II University in Naples). However, with a few exceptions, these centers are primarily concerned with systemic autoimmune diseases of rheumatological interest and focus mainly on research in order to develop innovative therapeutic solutions involving the immune system (human monoclonal antibodies, immune checkpoints inhibitors, probiotics) and to study their side effects [16–18]. Hence, management of autoimmune diseases for a holistic clinical view of these syndromes has not yet found wide application in hospitals around the world, partly disregarding the needs of patients often affected by autoimmune comorbidities [19].

However, we have to recognize that it is probably impractical to hypothesize a single clinical specialist be able to manage even a part of the many organ-specific and systemic autoimmune diseases. The initiatives described above therefore represent the result of very special conditions that originated in particularly favorable settings and therefore are not so easily reproducible in a widespread manner.

## The key role of the laboratory autoimmunologist

On the other hand, as we predicted eight years ago [12], we have seen the widespread creation of autoimmunology units within the clinical general laboratories in several parts of the world. In Italy as well as in other countries this phenomenon has materialized in parallel with the progressive transfer of autoimmune diagnostics from small-medium size hospital laboratories spread throughout the territory to big laboratories at the provincial/regional level. This epochal change to a model of consolidated analytical activity was made both for reasons of economies of scale and in order to concentrate professional expertise [10]. It also involved small but highly specialized university laboratories in moving towards a model of laboratory medicine less oriented to the academy and more to patient’s care [20]. This change has been greatly facilitated by the introduction of automation, thanks to the availability of stand alone or integrated analytical systems for higher throughput [21].

The introduction of paths for accreditation to excellence of clinical laboratories according to ISO 15,189 standards [22] also involving the sector of autoimmunology, has asked for new operational skills and new responsibilities of the laboratory autoimmunologist for active participation in patient care **[**23, 24]. Among these, management and technical requirements to control analytical processes, procedures for continuous quality improvement, definition of reference intervals, actions for correcting errors, reporting of laboratory results, definition and measurement of clinical outcomes, and availability of a consultant advisory service in the clinical-laboratory interface.

### The choice of diagnostic method for antibody detection

The focal point in the initial phase of the clinical-laboratory interface can be summarized as follows: the clinician requests the test, the laboratory autoimmunologist decides which method to use to perform the test [25]. This goal is usually accomplished by using first a screening method followed by identification of the responsible antibody (or antibodies) by specific methods. If available, methods that may enable the simultaneous application of antibody screening and identification can be used.

The decision on which analytical method is preferable for determining each antibody or groups of antibodies associated with any specific autoimmune disease therefore requires a thorough knowledge of all the characteristics of that particular antibody (i.e., isotype, antigenic epitope composition, prevalence, clinical association) and of the analytical methods (i.e., sensitivity, specificity, imprecision, and predictive value).

For example, as regards the request for ANA for rheumatic diseases, the decision depends on the type of clinical suspicion: if systemic lupus erythematosus is suspected, a screening method in IIF on HEp-2 or an equivalent method can be used [26]. A positive result in the screening should be followed by the identification of the antibody in question by individual or multiparametric methods.

If the suspect is Sjögren’s syndrome, the combination of IIF and solid-phase assay is a better choice for detecting anti-Ro antibodies which can escape detection if only IIF is used [27]. In the case of idiopathic inflammatory myopathies it is almost mandatory to rely on immunoassays capable of immediately identifying an enlarged profile of antibodies [28]. Finally, in systemic sclerosis, the difficulty of detecting anti-RNA polymerase III antibodies in IIF can be overcome by the use of methods that include the target antigen.

However, all this applies only if the test is requested for diagnostic purposes. For the monitoring of the evolution of the disease and of the efficacy of treatment, for those antibodies whose blood levels have been shown to correlate with the clinical course, it is always advisable to use quantitative immunometric methods capable of accurately measuring changes in antibody levels. A fundamental point is, therefore, that several analytical methods are available in the autoimmunity laboratory, to be used in different clinical situations and according to different needs. A recent national survey aimed at assessing the state of the art of Italian autoimmunology labs showed that 84% of labs use at least three different analytical methods to detect antibodies and 33% of labs use five or more methods (unpublished data).

But it is not enough to choose the right analytical method. Given the wide availability of commercial kits within the same method and their variable performance in terms of diagnostic accuracy, the duty of the laboratory autoimmunologist is to be able to orientate in choosing the best commercial kit among those available within every single method.

### Clinical interpretation and reporting of autoantibody testing

Interpreting the results of laboratory tests in the clinical context is a task that falls to the attending physician. However, to be interpreted correctly, some antibody tests require knowledge and skills not possessed by clinicians (with some exceptions). For example, how should a discrepant result for anti-tissue transglutaminase (tTG) antibodies and for anti-endomysial antibodies (EMA) be interpreted in a patient suspected to have celiac disease, considering that anti-tTG assays have higher sensitivity but lower specificity than EMA? In this case, an expert laboratory autoimmunologist who knows the pro and cons of the diagnostic methods he/she uses, who analyzes hundreds or even thousands of samples keeping the quality of the results under control and who is therefore able to detect the possible deviation of a result from the usual performance of the method, usually knows how to evaluate and manage controversial data much better than a clinician.

If results of autoantibody tests do not fit into a recognizable clinical context, the result are handled with care until it has been evaluated further, which can be done either by testing another specimen from the patient or by testing the same specimen using another assay to confirm or refute the result.

Antibody profiles analyzed by multiparametric methods merit a separate concern. In general, the overall specificity of these methods is lower than that of the methods that measure individual antibodies; in other words, the more antibodies are measured at the same time, statistically greater is the risk of false positives [29]. This, too, is a field in which the interpretative competence of the laboratory autoimmunologist is higher than that of the clinician and will further extend when cluster analysis provided by new proteomic-based microarray technology becomes available [30].

Special mention is deserved by the recent classification of the International Committee on Autoantibody Patterns (ICAP) HEp-2 patterns in the diagnosis of ANA-associated autoimmune diseases [31, 32]. This classification generates 29 different types of fluorescence patterns (AC 1-29), each of which is expected to be associated with a specific disease [33], that a laboratory autoimmunologist well-trained in recognition of ANA IIF nuclear, mitotic and cytoplasmic patterns may indicate to the clinician.

Lastly, the expression of antibody test results in terms of likelihood ratio has been proposed as a valid way to transform numerical data into clinical information [34, 35]. Though not very easy to do and not immediately feasible for many labs, this option could add value to laboratory reports and be highly appreciated by clinicians.

Taken as a whole, the evidence once again supports the vision of the laboratory as a medical discipline and not as a purely technological discipline, in which the advice of the laboratory autoimmunologist assumes a decisive weight in guaranteeing efficacy throughout the diagnostic process.

### A new approach towards the definition of reference values of autoantibody tests

The positive or negative result of a test depends largely on the definition of the cutoff value. Defining reference intervals has always been a challenge for the clinical laboratory, and the autoimmunology lab is no exception to this rule. To this end, the laboratory autoimmunologist may choose between direct and indirect methods. Direct methods for defining reference intervals for any given antibody refer to a prior selection of the reference sample population (this method is usually used by kit manufacturers), excluding individuals with subclinical pathologies, which in the case of AIDs are extremely frequent [36]. For autoimmune diseases, these methods are also disputed on the basis of disease prevalence that is 3–10 times higher in females than in males [37, 38], and therefore a separate reference value for the female population should also be included. For instance, in the case of autoimmune thyroid diseases (AITD), the higher frequency of these diseases in the female population raises the likelihood of including subclinical conditions or early-stage illnesses in the group of reference subjects. The use of indirect methods as a solution to overcome this problem has been adopted in some laboratories [39]. In indirect methods the reference subjects are not selected individually; on the contrary, the reference intervals for ‘health’-related subgroups are derived by statistical means from the total distribution of test results stored in the laboratory database. Indeed, one fact that is probably missed by many is that, due to the low pre-test probability of test requests (a greater number of subjects undergo antibody tests more to exclude rather than confirm an autoimmune disease), most of test results (in our experience between 90 and 95%) lay within the reference range. For this reason, the availability of vast amounts of digitized data in the Laboratory Information System makes it practicable today for clinical laboratories to use these methods that are better tailored for the local population. Indirect methods also allow the definition of different reference intervals based on age and gender that, for some antibodies, may be different between children and adults.

Of particular concern are the results which lie in a borderline zone. In such cases they may be reported with a caveat notice that the result must be interpreted with caution compared to a clear positive result; alternatively, another immunoassay can be used to confirm or refute the borderline result; or the cutoff can be set at a higher value to increase the diagnostic specificity. As a matter of fact, all these three options are used by the laboratory autoimmunologist on the basis of the clinical information and the performance characteristics of the assay used.

### The consultant advisory service of lab autoimmunologists

A very important phase of the relationship between clinical and laboratory autoimmunology is the vision of the laboratory autoimmunologist as a consultant to the clinician in improving the appropriateness in test request and in the interpretation and use of laboratory data [40], including guidance on follow up testing or even recommending course of action in selected cases [41]. This goal can be achieved through common guidelines or consensus documents, and by working jointly to disseminate recommendations for the rational use of antibody tests.

Due to the broadening spectrum of clinicians that use and rely on autoantibody testing in their practices,

the consultancy activity of the laboratory autoimmunologist finds its utmost utility in the relationship with non-autoimmunity specialists and general practitioners. A survey conducted in the US by the American Medical Association among general internal medicine and family physicians revealed that the percentage of the participants reporting that laboratory advice was very or extremely helpful ranged from 37% for assistance with appropriate test ordering based on patient’s symptoms and history to 65% for assistance regarding sample collection or submission and 71% for interpretative comments in the report [42].

In addition, given that a relevant feature of autoimmune diseases is their tendency to aggregate in multiple autoimmune syndromes (MAS) [43], the role of the laboratory autoimmunologist in managing unexpected results [44] and investigating this condition is clinically important, especially in the various forms of MAS type 3 [19, 45], that can escape recognition by the single clinical specialist. A case in point is the therapy of hypothyroidism in patients with AITD treated orally with l-thyroxine. Some patients may present a suboptimal response to treatment, due to concomitant autoimmune gastritis that occurs in 25% of AITD patients [46]. This clinical information transferred to the laboratory autoimmunologist may activate detection of autoantibodies to gastric mucosa or to intrinsic factor which may give a hint regarding therapy failure.

Lastly, an important issue is analytical interferences. Though their contribution to analytical errors is very small (0.078% of overall laboratory errors) [47], clinicians receive the results of the laboratory confident that they are as accurate as possible. When immunoassays are considered, interferences may be caused by lipemia, hemolysis or icterus that can be easily detected before starting the analytical process, or they may be due to unusual constituents present in the serum of individual subjects, not detectable before the analysis [47]. In particular, interferences may affect some immunoassays (heterophilic antibodies, human anti-murine antibodies, monoclonal gammopathies, immunocomplexes, rheumatoid factors, etc.) [48, 49], and some analytical platforms (e.g. assays based on biotin-streptavidin principles) [50]. This situation needs continuous communication and exchange of information between clinicians and laboratory scientists in order to minimize the risk of clinical mishandling arising from erroneous analytical results. This can be achieved mainly by informing clinicians when immunoassay results may be particularly vulnerable to interference, and always encouraging them to question unexpected results.

Based on awareness that only a close collaboration between the clinic and the laboratory can ensure the best outcome for the patient, recently a working group of the International Federation of Clinical Chemistry has proposed that consultancy should constitute a parameter of judgment on the performance of the Laboratory, included among the quality indicators [51].

## Conclusions

While the presence of autoimmunology units in hospitals has only been realized in part, the role and tasks of laboratory autoimmunologists have greatly expanded in the last 10–15 years, to the point that they represent a key factor to provide an answer to the increasingly emerging clinical needs and patients’ expectations. The work of a laboratory autoimmunologist therefore does not end with guaranteeing state-of-the-art analytical performance but now includes determining a better outcome for patients by working closely with hospital and family doctors.

## Data Availability

Not applicable.
